# FatPlants: a comprehensive information system for lipid-related genes and metabolic pathways in plants

**DOI:** 10.1093/database/baae074

**Published:** 2024-08-05

**Authors:** Chunhui Xu, Trey Shaw, Sai Akhil Choppararu, Yiwei Lu, Shaik Naveed Farooq, Yongfang Qin, Matt Hudson, Brock Weekley, Michael Fisher, Fei He, Jose Roberto Da Silva Nascimento, Nicholas Wergeles, Trupti Joshi, Philip D Bates, Abraham J Koo, Doug K Allen, Edgar B Cahoon, Jay J Thelen, Dong Xu

**Affiliations:** Institute for Data Science and Informatics, University of Missouri, 22 Heinkel Building, Columbia, MO 65211, United States; Christopher S. Bond Life Sciences Center, University of Missouri, 1201 Rollins St, Columbia, MO 65211, United States; Christopher S. Bond Life Sciences Center, University of Missouri, 1201 Rollins St, Columbia, MO 65211, United States; Department of Electrical Engineering and Computer Science, University of Missouri, Lafferre Hall, 416 S 6th St, Columbia, MO 65201, United States; Christopher S. Bond Life Sciences Center, University of Missouri, 1201 Rollins St, Columbia, MO 65211, United States; Department of Electrical Engineering and Computer Science, University of Missouri, Lafferre Hall, 416 S 6th St, Columbia, MO 65201, United States; Christopher S. Bond Life Sciences Center, University of Missouri, 1201 Rollins St, Columbia, MO 65211, United States; Department of Electrical Engineering and Computer Science, University of Missouri, Lafferre Hall, 416 S 6th St, Columbia, MO 65201, United States; Christopher S. Bond Life Sciences Center, University of Missouri, 1201 Rollins St, Columbia, MO 65211, United States; Department of Electrical Engineering and Computer Science, University of Missouri, Lafferre Hall, 416 S 6th St, Columbia, MO 65201, United States; Christopher S. Bond Life Sciences Center, University of Missouri, 1201 Rollins St, Columbia, MO 65211, United States; Department of Electrical Engineering and Computer Science, University of Missouri, Lafferre Hall, 416 S 6th St, Columbia, MO 65201, United States; Christopher S. Bond Life Sciences Center, University of Missouri, 1201 Rollins St, Columbia, MO 65211, United States; Department of Electrical Engineering and Computer Science, University of Missouri, Lafferre Hall, 416 S 6th St, Columbia, MO 65201, United States; Christopher S. Bond Life Sciences Center, University of Missouri, 1201 Rollins St, Columbia, MO 65211, United States; Department of Electrical Engineering and Computer Science, University of Missouri, Lafferre Hall, 416 S 6th St, Columbia, MO 65201, United States; Christopher S. Bond Life Sciences Center, University of Missouri, 1201 Rollins St, Columbia, MO 65211, United States; Department of Electrical Engineering and Computer Science, University of Missouri, Lafferre Hall, 416 S 6th St, Columbia, MO 65201, United States; Christopher S. Bond Life Sciences Center, University of Missouri, 1201 Rollins St, Columbia, MO 65211, United States; Department of Electrical Engineering and Computer Science, University of Missouri, Lafferre Hall, 416 S 6th St, Columbia, MO 65201, United States; Christopher S. Bond Life Sciences Center, University of Missouri, 1201 Rollins St, Columbia, MO 65211, United States; Department of Biochemistry, University of Missouri, Schweitzer Hall, 117, 503 S College Ave, Columbia, MO 65211, United States; Christopher S. Bond Life Sciences Center, University of Missouri, 1201 Rollins St, Columbia, MO 65211, United States; Department of Electrical Engineering and Computer Science, University of Missouri, Lafferre Hall, 416 S 6th St, Columbia, MO 65201, United States; Institute for Data Science and Informatics, University of Missouri, 22 Heinkel Building, Columbia, MO 65211, United States; Christopher S. Bond Life Sciences Center, University of Missouri, 1201 Rollins St, Columbia, MO 65211, United States; Department of Electrical Engineering and Computer Science, University of Missouri, Lafferre Hall, 416 S 6th St, Columbia, MO 65201, United States; Department of Biomedical Informatics, Biostatistics and Medical Epidemiology, University of Missouri, CE707, Clinical Support and Education Building, 5 Hospital Dr. Columbia, MO, United States; Institute of Biological Chemistry, Washington State University, 101D Plant Sciences Building, Pullman, WA 99164, United States; Department of Biochemistry, University of Missouri, Schweitzer Hall, 117, 503 S College Ave, Columbia, MO 65211, United States; Agriculture Research Service, United States Department of Agriculture, 975 N Warson Rd, St. Louis, MO 63132, United States; Donald Danforth Plant Science Center, 975 N Warson Rd, St Louis, MO 63132, United States; Department of Biochemistry and Center for Plant Science Innovation, University of Nebraska, 1901 Vine St, Lincoln, NE 68588, United States; Christopher S. Bond Life Sciences Center, University of Missouri, 1201 Rollins St, Columbia, MO 65211, United States; Department of Biochemistry, University of Missouri, Schweitzer Hall, 117, 503 S College Ave, Columbia, MO 65211, United States; Institute for Data Science and Informatics, University of Missouri, 22 Heinkel Building, Columbia, MO 65211, United States; Christopher S. Bond Life Sciences Center, University of Missouri, 1201 Rollins St, Columbia, MO 65211, United States; Department of Electrical Engineering and Computer Science, University of Missouri, Lafferre Hall, 416 S 6th St, Columbia, MO 65201, United States

## Abstract

FatPlants, an open-access, web-based database, consolidates data, annotations, analysis results, and visualizations of lipid-related genes, proteins, and metabolic pathways in plants. Serving as a minable resource, FatPlants offers a user-friendly interface for facilitating studies into the regulation of plant lipid metabolism and supporting breeding efforts aimed at increasing crop oil content. This web resource, developed using data derived from our own research, curated from public resources, and gleaned from academic literature, comprises information on known fatty-acid-related proteins, genes, and pathways in multiple plants, with an emphasis on *Glycine max, Arabidopsis thaliana*, and *Camelina sativa*. Furthermore, the platform includes machine-learning based methods and navigation tools designed to aid in characterizing metabolic pathways and protein interactions. Comprehensive gene and protein information cards, a Basic Local Alignment Search Tool search function, similar structure search capacities from AphaFold, and ChatGPT-based query for protein information are additional features.

**Database URL**: https://www.fatplants.net/

## Introduction

Vegetable oils are an energy-dense renewable feedstock for chemicals and fuels and are an essential component of the human diet [1]. It is estimated that by 2050, the current vegetable oil production will need to double to meet societal needs [2, 3]. To date, increases in plant seed oil production through engineering or breeding have been reported but often failed to meet expectations [3–7]. Such efforts have often resulted in unintended consequences, including reduced seed shelf life and germination rate, and adverse effects on negatively impacted protein content. Lipid metabolism is a highly branched metabolic network that produces both membrane lipids and storage oils [2, 3, 8], and takes place across multiple organelles [9]. The regulatory nodes and metabolic bottlenecks [10, 11] that affect seed oil and protein accumulation are only partially characterized at the genetic and biochemical levels [8]. Hence, improving plant seed oil will require extensive effort. Such a challenge would benefit from a web portal equipped with analysis and visualization tools for fatty-acid-related proteins, which would comprehensively archive data and accelerate the process of knowledge discovery and crop design for biologists. Easy access to built-in analysis tools is also needed to empower researchers to develop and test hypotheses and design crops with value-added compositions.

Web resources are starting to emerge that have been developed to describe plant acyl-lipid metabolism or curate fatty-acid-related data, but frequently they are limited in scope and out of date. For example, Lipidbank [12], Seed Oil Fatty Acids Database [13], and LIPIDAT [14] are no longer maintained or updated; ARALIP [8], a widely used plant lipid-related protein database, focuses on *Arabidopsis thaliana* only; LIPIDMAPS [15] lacks integrated pathway knowledge; PlantFAdb [16] and Plant Lipid Databases [17] concentrate on the chemophysical properties and structures of lipids only. The growing research needs in plant lipids call for the development of a new platform that can provide comprehensive coverage of oilseed plants, genes, and knowledge in this area, and can continue to grow and improve with facile incorporation of community input.

To assist researchers in studying plant fatty acid metabolism efficiently, we developed a one-stop-shop web resource, FatPlants. Protein data has been manually curated and entered relevant to fatty acid metabolism in *Glycine max* (soybean), *A. thaliana* (Arabidopsis), and *Camelina sativa* (Camelina) from Uniprot [18], TAIR [19], SoyKB [20], KBcommons [21] LIPIDMAPS [15, 22], PlantFAdb [16], CamRegBase [23], and ARALIPS [8]. Molecular information on the fatty acid composition, chemical structures, and chemophysical properties from OPSIN [24] provides an in-depth description. For each protein record, general annotations from UniProt [18], including postmodification regions or sites, have been collected. For each specific species, we have included the cross-linked identifiers for different databases and the external links so that users can easily redirect to those databases. Sequences, annotation, and description are provided together with the structure information of those fatty-acid-related proteins.

Following data curation, we established a user-friendly searchable database augmented with visualization tools. FatPlants offers a suite of analysis features, including sequence or structure similarity searches. Users can submit a protein sequence to our database and obtain a list of similar proteins. Alternatively, the structure similarity method allows users to provide a protein sequence, and FatPlants returns proteins with the most analogous 3D structures based on AlphaFold API [25]. Functional analysis is facilitated by mapping fatty-acid-related proteins to pathway databases. We have manually converted images of fatty-acid-related pathways from academic literature into interactive graphs using machine learning, enabling users to explore protein or gene elements of lipid metabolism in depth. This feature allows regular updates with the latest fatty-acid-related pathways from recent literature. FatPlants provides links to protein–protein interaction (PPI) and Gene Ontology (GO) enrichment networks for fatty-acid-related genes. A unique feature we have integrated into FatPlants is the utilization of the ChatGPT API, enabling users to obtain specific protein information interactively. In essence, FatPlants serves as a comprehensive platform for plant fatty-acid-related data, knowledge, and analysis, with user-friendly search and analysis tools to facilitate understanding of the underlying biological frameworks.

## Materials and methods

### Data acquisition and curation

**Figure 1.**
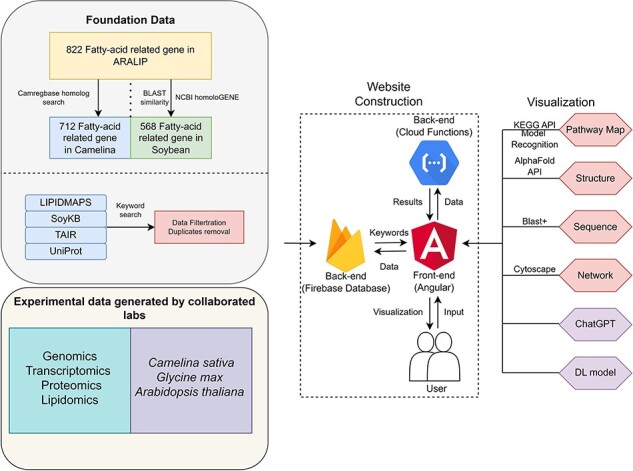
The data schema and functionalities of FatPlants.

Our datasets were collected from three primary data sources, described in Fig. 1: ARALIP centered data, searchable data from protein databases, and in-house and published experimental data. The ARALIP data containing fatty acid-centric enzyme/gene data from Arabidopsis was utilized by searching homologs in Camelina and soybean, resulting in 712 genes in Camelina and 568 in soybean. We searched for proteins in the UniProt database with keywords including ‘lipid’ and ‘fatty acid’ in three species: G. max, A. thaliana, and C. sativa, and conducted the same keyword search in the TAIR database. For Arabidopsis, we collected 2447 fatty-acid-related proteins of Arabidopsis from LIPIDMAPS. Regarding physical and chemical properties data, we have collected 495 entries from PlantFAdb. The keywords ‘lipid’ and ‘fatty acid’ were used to search for genes with UniProt and the soybean Gene Model V9.00 in SoyKB. To perform data filtration, we mapped all the identifiers to UniProt ID and removed redundant and unannotated proteins. A total of 3440 fatty-acid-related proteins were obtained for Arabidopsis and 5606 for soybean (Table 1). Fatty-acid-related protein data for Camelina could not be collected due to a lack of annotation. Therefore, a homology search against CamRegBase was performed to find the fatty-acid-related proteins of Camelina by using Arabidopsis data.

**Table 1.** A summary of acyl-lipid metabolism data collection from different databases

	*Arabidopsis thaliana*	*Camelina sativa*	*Glycine max*
ARALIP centered	822	712	6602
UniProt	1559	223	422
TAIR	1718	N/A	N/A
SoyKB	N/A	N/A	389
LIPIDMAPS	2447	N/A	N/A
CamRegBase	N/A	9810	N/A
Total (Filtered)	6546 (3440)	10 845 (8581)	7413 (5606)

The total number represents the raw data we collected from the database source, and the filtered number shows how many proteins are left after our filtration schema. The N/A indicates that species are unavailable in the specific database.

The protein list was used to retrieve the structure data from the Research Collaboratory for Structural Bioinformatics Protein Data Bank [26]. The PPI data were collected from the STRING database [27] and visualized in networks by direct or indirect interactions with intermediate nodes. The GO hierarchical annotations were retrieved from the GO [28] database and enriched and visualized in the network. Fatty-acid-related proteins were mapped to the Kyoto Encyclopedia of Genes and Genomes (KEGG) pathway database. In addition, a collection of fatty-acid-related pathway pictures from the literature [29–33] were visualized as an interactive map using our in-house machine-learning image understanding tool [34].

### Database and web interface implementation

FatPlants provides a user-friendly interface for data access and retrieval. It is implemented by a frontend Single-Page-Application architecture using Angular 10.0. The application interacts with users dynamically to update the current web page. In the backend, we have developed a document-oriented database based on Firestore 9.1.3. As shown in Fig. 1, the entire dataset is stored in Firebase with extensive authentication and a dynamic log system. FatPlants is deployed on Firebase. All backend functions associated with the Linux environment or outside APIs were implemented as Google Cloud Functions to accelerate the response time and reduce server latency. For feature development, the JavaScript library of Cytoscape [35] was used to visualize all network data and the Linux version of Basic Local Alignment Search Tool (BLAST) to build a sequence search function. In an innovative approach to enhance the accessibility of information, we integrated the ChatGPT API into our platform. This allows users to interactively retrieve specific protein information using natural language queries, thereby simplifying the process of data mining. Previously developed tools for structural prediction and pathway image recognition were incorporated to enable lipid characterization [25, 34]. In addition, to provide a smooth user experience of usage, FatPlants was validated on different browsers, such as Google Chrome, Edge, and Safari. It is also suitable for iOS and Android mobile devices.

## Results

As an overview of the main content at our site, we include 2341 acyl-lipid metabolism proteins for *A. thaliana*, 1232 for *G. max*, and 623 for *C. sativa*. These data have extensive information about their properties, functions, descriptions, and modification domains. Chemical information is provided for a total of 495 fatty acids. Twelve PPI networks of Arabidopsis and 10 GO-enrichment networks can be visualized based on different metabolic pathways. Currently, 15 auto-recognized pathways have been retrieved from the latest published papers. Since the search function is linked to the KEGG and Protein Data Bank databases, users can study additional data via FatPlants.

### Web interface and usage

**Figure 2.**
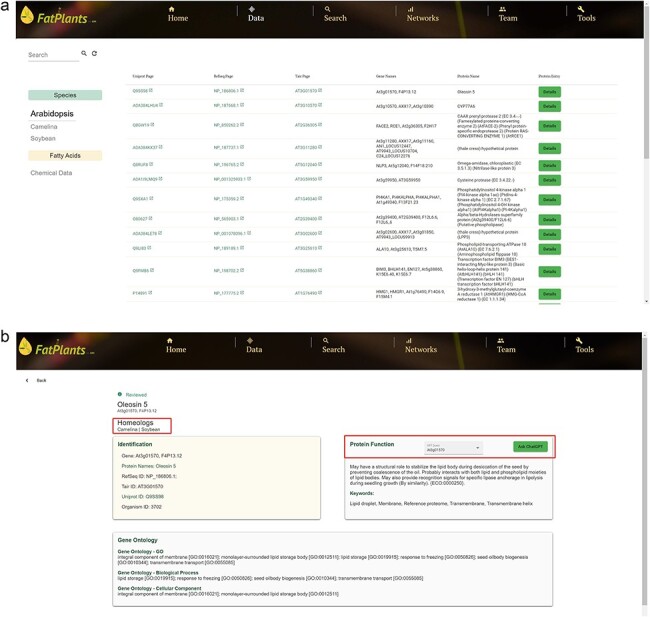
FatPlants data browse page. (a) The main data browse table. (b) The information card page for a selected protein with homologs and ChatGPT features.

FatPlants offers a user-friendly web interface, enabling users to conveniently browse, search, and retrieve data on fatty-acid-related proteins. Six functional header menus are situated on the top navigation bar—‘Home’, ‘Data’, ‘Search’, ‘Networks’, and ‘Tool’—designed to facilitate easy access to the database. The ‘Home’ page provides a concise overview of our database and its three primary functions. Users can explore the principal datasets via the ‘Browse’ menu. On the main data page (Fig. 2a), FatPlants offers a selection panel for users to switch between species and fatty acids. Leveraging the Angular framework, we developed an instant filter search function within data tables. Users can search for any protein by submitting identifiers, gene names, or gene descriptions. Each protein is linked to its corresponding database using unique identifiers. For every specific protein, we provide a detailed information page encompassing key identifiers, functional annotation, functional sequence domain, and the protein function description. The ‘Ask ChatGPT’ button offers an additional avenue for users seeking in-depth knowledge about a specific protein. Furthermore, the ‘Homologs’ section indicates related homologous proteins in other species (Fig. 2b).

### One-stop search

To accommodate the possibility that proteins might have multiple identifiers (UniProt ID, RefSeq ID, etc.), we have constructed an internal identifier mapping dictionary. This dictionary incorporates seven classes of widely used IDs: UniProt ID, Protein Name, Gene Symbol, EMBL ID, EnsemblPlants ID, STRING ID, and Locus ID. Any identifier entered will automatically link to a specific protein in our database. The core analysis features of FatPlants include a one-stop-search function based on a sequence similarity search, similar to BLASTP, and a structure similarity search algorithm utilizing the AlphaFold API. Users can effortlessly search for a given protein against the FatPlants fatty-acid-related protein database to find similar sequence or structure results and visualize them in a 3D model (Fig. 3). Moreover, a pathway mapping function is available through the KEGG API. As depicted in Fig. 3a, the one-stop search function accepts both sequences and identifiers as input. The default page displays a summary result, including the most structurally similar 3D model, identifier list, and sequence. Users can toggle between three different result types from the side panel. Figure 3b presents a structure similarity result table generated by Alphafold API. The Blast results display all candidate matches from the FatPlants data collection (Fig. 3c). On the pathway mapping result page, graphs depict all pathways involving the input protein (marked by the red boxes), as provided by the KEGG API (Fig. 3d).

**Figure 3.**
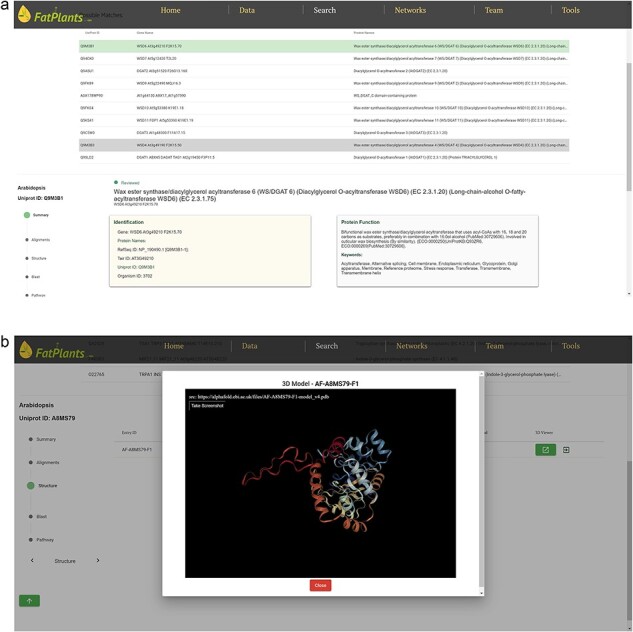
One-stop search page of FatPlants. (a) The summary result page with all the candidates (in this case, we use name DGAT). (b) The structure result page, which is retrieved from Alphafold API. (c) The BLASTP result page. (d) KEGG pathway mapping page with the target gene highlighted in the red box.

**Figure 3.**
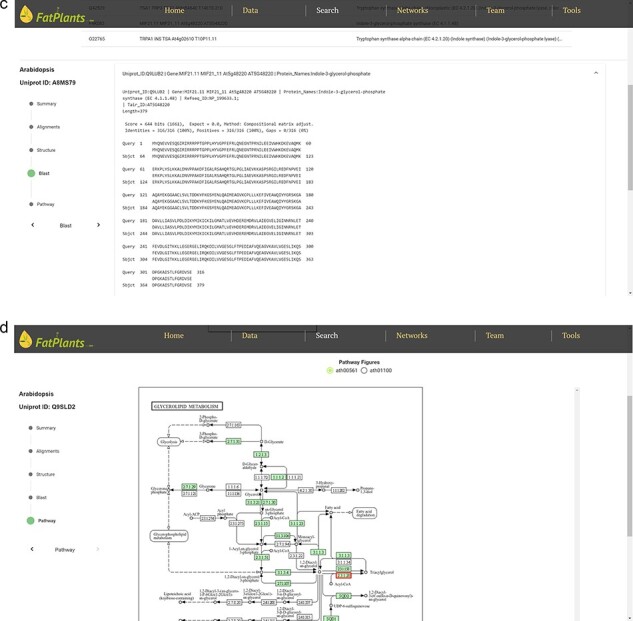
(Continued)

### Network viewers

**Figure 4.**
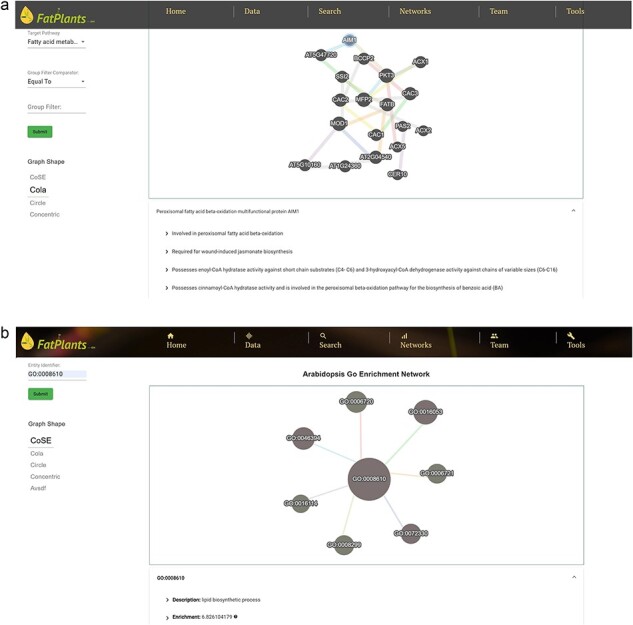
The FatPlants network viewer. (a) PPI network related to the fatty acid metabolism pathway. (b) GO enrichment network which includes gibberellin-related terms.

The proteins in FatPlants can also be visualized in the context of PPI networks based on the STRING database [27]. We present all PPI networks in terms of their locations within the metabolic network. Figure 4a provides a PPI example in the fatty acid metabolism category. Users can easily browse the PPI network by selecting the desired pathways and clicking a network node to explore the protein’s functional description in the bottom table. The fatty-acid-related protein GO enrichment network can be visualized through an enrichment network page to capture the enrichment connection between ontology terms. Users can search any specific protein using different identifiers to retrieve the ontology information. An example of a lipid biosynthetic process involving seven other GO terms enrichment (monocarboxylic acid biosynthetic process, isoprenoid metabolic process, organic acid biosynthetic process, carboxylic acid biosynthetic process, terpenoid biosynthetic process, isoprenoid biosynthetic process, and terpenoid metabolic process) is presented in Fig. 4b as an example.

### Custom pathway viewer

**Figure 5.**
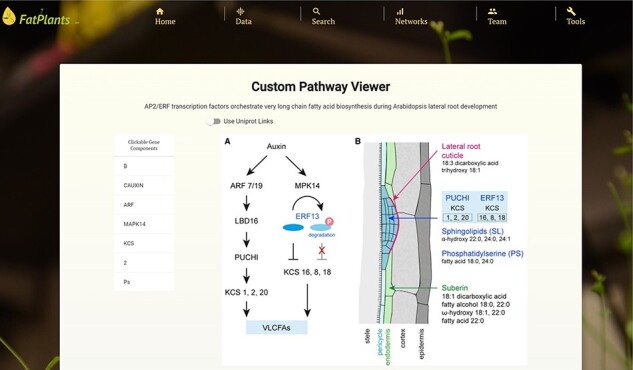
An example of Custom Pathway Viewer on FatPlants. The original graph is from ‘Expression of sets of VLCFA biosynthetic genes is regulated by AP2/ERF transcription’ [31].

**Figure 6.**
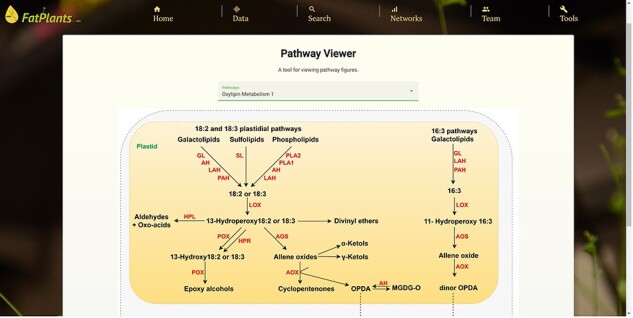
An example of a manually drafted pathway (oxylipin metabolism pathway).

Within the custom pathway viewer page, users can manually submit pathway graphs from fatty-acid-related research papers. Leveraging our in-house machine learning image understanding tool [34], these submitted pathway graphs are transformed into interactive pathway maps, where genes/proteins are linked to entries in FatPlants. We currently showcase 15 graphs as trial datasets [29–33]. Figure 5 provides an example of this tool’s functionality. Protein elements that can be interacted with are highlighted in red when hovered over, and all recognized proteins are cataloged in a table on the page. Users can access detailed information from the FatPlants database or the comprehensive protein records in the UniProt database. This tool enables FatPlants to integrate the latest fatty acid pathway research, capturing key interactions with crucial proteins. In addition to the machine learning-based pathway graphs, we have a set of manually drafted pathway graphs. It presents a graphical representation inspired by ARALIP [8] (Fig. 6).

### A use case example

**Figure 7.**
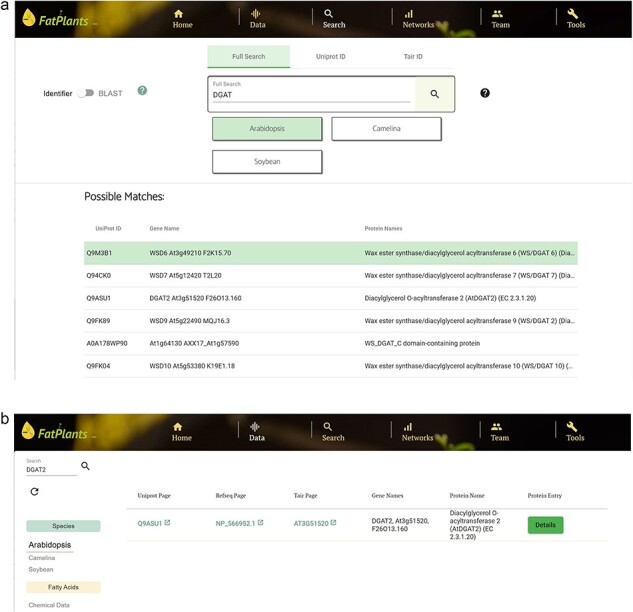
A use case for searching diacylglycerol O-acyltransferase 2, with gene symbol DGAT2. (a) Match results using ‘DGAT’ as the input on the ‘Search’ page. (b) Search result for ‘DGAT2’ on the ‘Data’ page by selecting the target species (Arabidopsis).

Diacylglycerol O-acyltransferase 2 (gene symbol DGAT2) is involved in triacylglycerol synthesis. It catalyzes the acylation of the sn-3 hydroxy group of sn-1,2-diacylglycerol using acyl-CoA. To find related information on this gene, a user can perform a partial search on the ‘Search’ page by entering ‘DGAT’ to obtain a list of hits, as shown in Fig. 7a. The user can select a hit of interest to explore more information, such as protein structure and similar sequences. The user can also search for DGAT2 on the ‘Data’ page by selecting the target species (Arabidopsis in this case), which leads to a unique hit, as shown in Fig. 7b.

## Conclusions and future work

FatPlants is a comprehensive and systematic fatty-acid-related protein database resource. It can help users understand plant oil synthesis and breeders improve oil content. Users can also leverage AI assistance to gain deeper insights into specific proteins. FatPlants provides several network-based data representations and visualization tools to explore fatty-acid-related protein functions and relationships. By integrating different tools, the one-stop search can help users retrieve the corresponding information efficiently and comprehensively.

For future work, this data repository and a suite of visualization and analysis tools will be continuously updated with new data collected from oilseed research, particularly for important emerging crops such as Camelina and pennycress, two related Brassicaceae species that are not as well-developed as Arabidopsis. User feedback will guide new analysis or visualization tools to explore the fatty-acid-related protein data. To take advantage of our in-house image understanding tool, a Web-based pipeline will be developed for users to submit fatty-acid-related pathway figures. The pipeline will automatically parse the figures into pathway components and their relationships. In addition, we are implementing an internal API to collect the latest plant lipid publications on PubMed so that FatPlants can be updated accordingly. We will also use some large language models, such as ChatGPT, to help identify more relevant data/knowledge sources for FatPlants.

## Data Availability

The FatPlants database is publicly available at https://www.fatplants.net/.
